# Perception of the Residential Living Environment: The Relationship Between Objective and Subjective Indicators of the Residential Living Environment and Health

**DOI:** 10.3390/ijerph22030391

**Published:** 2025-03-07

**Authors:** Joachim Gotink, Sylvie Gadeyne

**Affiliations:** Department of Sociology, Vrije Universiteit Brussel, Pleinlaan 2, 1050 Brussels, Belgium; sylvie.gadeyne@vub.be

**Keywords:** mortality, death, environmental health, health inequalities and air pollution

## Abstract

Multiple studies have found an association between ambient air quality, noise pollution, green spaces and health. The underlying mechanisms of this association remain partly unknown. In this study, we focus on subjective perception as a potential underlying factor. We assess (I) the association between objective and subjective indicators of the living environment and all-cause mortality, as well as (II) the potential modification of the relationship between objective exposures and all-cause mortality by subjective perception: The data consisted of a linkage between the 2001 census, mortality register data from 1 October 2001 to 31 December 2016 and objective indicators of the residential living environment (air and noise pollution and green spaces). We used Cox regression to investigate the impact of objective and subjective indicators of the living environment and their potential interaction effect on all-cause mortality in the Brussels Capital Region: A negative subjective perception of the residential living environment is associated with an increased risk of mortality, even when controlling for socio-demographic parameters. Similarly, objective indicators of air pollutants and green spaces are also related to mortality. When studying the interaction effect, the beneficial effect of a neutral subjective perception stands out. Subjectively satisfied individuals living in the worst objective conditions showed the highest level of mortality hazard. Noise pollution was the only exception, characterized by the lack of an interaction effect: This study showed that besides objective indicators, the subjective perception of the residential environment also matters, and both interact to influence life chances. Subjective indicators not only have a genuine independent impact but also act as an underlying factor in the relationship between the objective residential environment and health.

## 1. Introduction

The link between environmental factors and health is well established. Numerous studies have shown that exposure to air pollution is associated with severe health outcomes, including an increased incidence of lung cancer in adults [1], impaired lung development in children [2] and a higher risk of premature births [3]. Similarly, noise pollution has been linked to an elevated risk of cardiovascular diseases [4], increased incidences of diabetes [5] and an increased prevalence of isolated systolic hypertension [6]. However, long-term exposure to noise pollution does not appear to be significantly associated with all-cause mortality [7]. Increasing evidence suggests that green spaces have a beneficial impact on well-being, including self-reported health [8], lower overall mortality rates [9,10] and potential benefits for individuals with chronic heart failure [11].

Despite these well-documented associations, the underlying mechanisms remain only partially understood. Of course, there are the biological effects of these exposures. For example, the biological pathogenesis of exposure to PM_2.5_ (particulate matter with a diameter smaller than 2.5 µm) has been clearly described in the literature, where it is associated with inflammation, oxidative stress and a potential influence on epigenetic regulation [12]. Similarly, noise pollution has been linked to a disruption of the circadian rhythm (which increases the risk for a multitude of adverse health outcomes) and an in increase in several inflammatory markers [13]. While biological mechanisms are important, subjective perceptions of environmental factors can also play a critical role in shaping health outcomes by potentially mitigating the biological impact of environmental exposure. This is shown by theories such as the stress reduction theory and attention restoration theory [14]. Lachowycz et al. (2013) found that the subjective perception of green spaces can modify the observed effect of green spaces on health outcomes [15]. Similarly, Babisch et al. (2013) found that the association between objective noise measurement (near airports) and hypertension was stronger for people who reported being more subjectively annoyed by aircraft noise pollution [16]. The influence of subjective perception could also be interpreted through Lazarus’s (1984) transactional model of coping and stress [17]. Lercher (1996), who further discussed this model, stated that perception or appraisal is a process through which a person evaluates an exposure. If an individual is aware of a stressful exposure, this might lead to the adaptation of cognitive, behavioural and emotional coping mechanisms [18]. A recent study in Kenya, for instance, demonstrated that even the mere participation in air quality studies led residents to develop strategies for coping with air pollution in their neighbourhood [19].

Our study aims to address this gap in the literature by using the Brussels Capital Region (BCR) as a case study. The BCR is the largest metropolitan area in Belgium, with clear divisions in environmental exposures and socio-economic variables, making it a good setting to investigate these complex relationships. The primary objectives of this paper are twofold: it aims (i) to examine the association between objective and subjective perceptions of the residential (outdoor) living environment (including air and noise pollution and green space exposure) and all-cause mortality, and (ii) to explore how subjective perception might modify the relationship between objective indicators of the living environment and all-cause mortality. By addressing these questions, this study seeks to contribute new insights into how environmental factors, both perceived and measured, may impact long-term health outcomes in urban settings.

## 2. Methodology

The data consist of a linkage between the Belgian 2001 census—including detailed information on the socio-economic and demographic characteristics and subjective indicators of the residential living environment—and register data on mortality (and emigration) for the follow-up period spanning 1 October 2001 until 31 December 2016. The data were further linked to objective residential environmental indicators using the residential address of each individual at baseline. We included all persons aged 25–79 officially residing in the Brussels Capital Region at baseline (1 October 2001).

Our study focused on three environmental dimensions: ambient air pollution, noise pollution and green spaces. The objective indicators included estimates of air and noise pollution levels and a green density metric derived from satellite images. Ambient air pollution was measured using the 2005 average annual concentrations (µg/m^3^) of nitrogen dioxide (NO_2_) and particulate matter with an aerodynamic diameter of less than 2.5 µm (PM_2.5_) (Belgian Interregional Environment Agency (IRCEL)). Noise pollution was operationalized by combining noise levels from air, road and railway traffic into one indicator expressed as the 24 h average [day–evening–night (L_den_)] noise level in decibels (dB(A)) (Environment.Brussels). The surrounding greenness was assessed using the Normalized Difference Vegetation Index (NDVI). The NDVI ranges from 0 to 1, with values closer to 1 indicating greater green density [20]. A more in-depth description of these indicators is available in the study by Rodriguez-Loureiro et al. [21].

NO_2_ concentrations (threshold of 40 µg/m^3^) and noise pollution (53 dB(A) L_den_ threshold) were dichotomized according to the WHO threshold (below/above WHO threshold). For PM_2,5_ concentrations, we used quintiles since all values exceeded the 10 µg/m^3^ annual average WHO threshold in Belgium. Surrounding greenness was also categorized using quintiles as WHO does not define any thresholds for this variable.

The subjective perception of the living environment was derived from the 2001 Belgian census. For each household, respondents were asked to indicate how they perceived the air quality, noise pollution and surrounding greenness in their neighbourhood on a 3-point Likert scale (satisfied, neutral or not satisfied).

To investigate potential interaction effects between subjective and objective indicators of the living environment and all-cause mortality, we cross-classified both variables. Our reference category consisted of the combination of a satisfied subjective perception with the lowest concentration of air pollutants, the lowest amount of noise pollution and the highest amount of surrounding greenness. All categories contained more than 1.0% of the cases.

We included gender, age, migration background (Belgian and non-Belgian), household living arrangement (couple, single, other), highest educational level (tertiary, secondary, primary) and housing tenure (owners, tenants, other) as control variables. The dependent variable consisted of all-cause mortality during the 15-year follow-up period.

We used Cox proportional hazard regression models to investigate the association between each dimension of the outdoor living environment and all-cause mortality. Similarly to Bauwelinck et al. (2021), we used age as an underlying time scale [9]. Observations were censored when emigration or end of follow-up occurred. To account for differential baseline hazards by age groups, we included a strata term with a 10-year categorization (e.g., from (25–30] to (30, 40] to (70, 80]).

**Figure 1:**
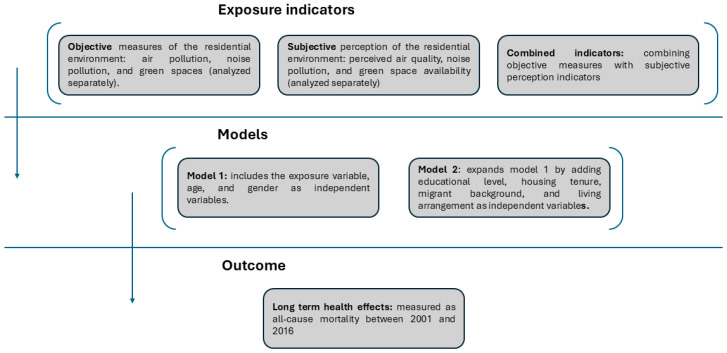
Overview of the study approach: associations between objective and subjective environmental indicators and all-cause mortality, analyzed using basic (M1) and fully adjusted (M2) models.

First, we assessed the association between each objective indicator (one at a time) and all-cause mortality. Second, we assessed the association between each subjective indicator (one at a time) and all-cause mortality. Finally, we used the combined indicator (objective + subjective) for each environmental dimension to assess the association with all-cause mortality. For each model, we specified a basic model M1 (including the exposure indicator, age and gender) and a fully adjusted model M2 (additionally adjusting for educational level, housing tenure, migrant background and household living arrangement). This workflow is also shown in Figure 1.

## 3. Results

Table 1 contains a detailed description of the study population at baseline (2001), all-cause mortality during follow-up and the objective and subjective indicators of the living environment. After excluding individuals with missing values (24.8%) for covariates, our study population consisted of 464,611 individuals aged 25–79 officially residing in the BCR at baseline (2001). During follow-up (2001–2016), 66,832 individuals died and 40,617 emigrated. The study population consisted mostly of women (52.3%), individuals with a secondary education (43.4%), individuals originating from Belgium (59.4%) and individuals living with a partner (61.5%).

The median exposure to average annual concentrations of air pollutants was 19.27 µg/m^3^ (IQR: 0.85) for PM_2.5_ and 38.94 µg/m^3^ (IQR: 6.27) for NO_2_; the daily average exposure to multiple sources of noise pollution was 49.87 dB(A) (IQR: 5.87); residential surrounding greenness within a 300 m buffer was 0.43 (IQR: 0.19).

Overall, respondents tended to report a neutral perception of air and noise pollution levels, as well as of the availability of green spaces in their neighbourhood. The largest proportion of individuals were neither satisfied nor dissatisfied with these factors, as reflected in their middle-category responses. However, a substantial proportion of individuals found the noise pollution levels unpleasant (35.4%), with air quality and the availability of green spaces also being perceived negatively by 29.4% and 24.3% of respondents, respectively.

Table 2 shows the results of the associations between the objective indicators and all-cause mortality. In the basic model, all indicators generate hazard ratios (HRs) in the expected direction, with lower levels of air and noise pollution and a higher density of surrounding greenness being associated with lower mortality. Controlling for socio-demographic variables (M2) attenuated the effects, but associations remained significant (e.g., the quintile with the highest PM_2.5_ concentrations still has an increased mortality hazard of 16% compared with the reference category), except for noise pollution. Considering surrounding greenness within 300 m of residence, the largest excess mortality was observed for the two quintiles with the lowest residential greenness [1.15% (95% CI: 1.117; 1.192) and 1.28% (95% CI: 1.232; 1.320)].

The associations between the subjective indicators of the living environment and all-cause mortality are displayed in Table 3. Overall, rating any dimension of the living environment positively or neutrally generated lower HRs. After adjustment for covariates, the difference between rating the environment positively or neutrally disappeared [e.g., for green spaces: normally equipped HR 0.91 (95% CI: 0.885, 0.936); very well-equipped HR 0.91 (95% CI: 0.880, 0.933)]. Rating the noise pollution in the neighbourhood as very pleasant was not significantly associated with a decreased risk of all-cause mortality.

**Figure 2:**
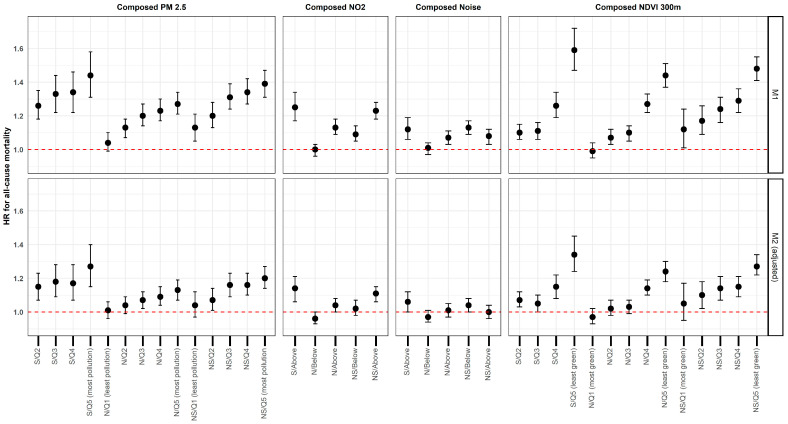
Hazard ratios (HRs) of all-cause mortality and 95% confidence intervals (95% CIs) for the composed variables of the residential living environment, BCR, 2001–2016. Abbreviations: Subjective perception: S = Satisfied, N = Neutral and NS = Not satisfied; objective indicators categorized into quintiles: Q1 = the most “optimal” quintile (i.e., least pollution, most surrounding greenness), … and Q5 = the least “optimal” quintile (i.e., most pollution, least surrounding greenness); objective indicators categorized based on WHO threshold: above = value is higher than the threshold value; below = value is lower than the WHO threshold value. Reference group: For each indicator, the reference group is the most “optimal condition” (i.e., a satisfied perception combined with the least pollution or most green space). Source: Belgian 2001 census linked to the mortality register (follow-up 1 October 2001–31 December 2016) and environmental exposure data.

Figure 2 presents the potential mediating effect of the subjective perception on the association between the objective residential living environment and mortality (results are fully reported in Tables S1–S3 in the Supplementary Material).

Overall, three general patterns emerged. Unexpectedly, individuals who reported being satisfied with their living environment appeared to be more strongly affected by poorer environmental conditions (such as higher levels of air pollution, greater noise pollution or less surrounding greenness). Specifically, the group that combined a satisfied subjective perception with the least favourable objective conditions had the highest hazard ratios for each exposure. For example, individuals in this category showed a hazard ratio of 1.34 [1.24; −1.45] for surrounding greenness.

Secondly, we observed a positive effect (i.e., a reduction in mortality hazard) associated with a neutral perception of air quality, noise pollution and surrounding greenness. Specifically, individuals who reported being either “very satisfied” or “not satisfied” significantly differed from the reference category when living in areas with a higher level of surrounding greenness (e.g., for the second quintile: S/Q2: HR 1.07 [1.03; 1.12]; NS/Q2: HR 1.10 [1.02; 1.18]). However, individuals with a neutral subjective perception did not show a significantly increased mortality hazard compared to the reference category when living in the second (HR 1.02 [0.98; 1.07]) or third quintiles (HR 1.03 [0.99; 1.07]) of surrounding greenness.

Thirdly, for individuals who were not satisfied with their residential environment, there was no substantial difference between the composed indicators (as shown above) and the objective indicators (Table 2), even though a negative perception itself led to higher hazard ratios for all-cause mortality (Table 3). In other words, for this specific group, there does not appear to be an additive effect between both the objective and subjective indicators. Otherwise, we would have expected this category to have worse results than the satisfied category, which is not the case.

Thirdly, there does not seem to be an additive effect of reporting poor environmental quality and being exposed to it. For the non-satisfied categories, the results for the objective indicators and composed indicators are rather similar, e.g., for surrounding greenness, an HR of 1.15 [1.12; 1.19] was observed for the fourth quintile. For the non-satisfied group of the composed variable, the HR was the same (HR 1. 15 [1.090; 1.210]) for this same quintile.

To strengthen our findings, we conducted several sensitivity analyses (See Tables S4–S6 in the Supplementary Material). First, we filtered out individuals that moved between 1991 and 2003 and repeated all analyses. The results for this subpopulation were in line with the results observed for the main population.

Secondly, we analyzed only the subpopulation that assessed its health as “good” or “very good” in the 2001 census. This approach was intended to partially address the lack of information on individual lifestyle factors (e.g., smoking, physical activity, etc.) that could influence all-cause mortality [22]. The influence of the objective indicators remained unchanged (i.e., higher levels of pollution and less surrounding greenness were still associated with a higher mortality hazard), but clear differences were observed with respect to the influence of the subjective indicators in comparison with the full population analysis. Specifically, individuals who were not satisfied with their living environment in terms of noise and air pollution exhibited significantly lower mortality hazards compared to the reference category (satisfied individuals), which also affected the results for the composed variables. However, individuals with a satisfied perception living in the least optimal objective conditions still had the highest mortality hazard.

## 4. Discussion

The results for the objective indicators align with the current literature. Higher concentrations of air pollutants [1,23] and less surrounding greenness [9,10,24,25] were significantly related to higher all-cause mortality hazard [21]. These findings persist even when controlling for socio-demographic variables. For noise pollution, we did not find a significant association with all-cause mortality. This result is consistent with Tonne et al. (2016) [7]. Other studies on noise pollution primarily focus on specific pathologies, which we could not verify in our study.

Babisch et al. (2013) found that a negative subjective perception (labelled “annoyance”) added to the risk of hypertension due to aircraft noise exposure [6]. This is a finding we could not replicate for all-cause mortality in our study. We found that mortality hazards obtained for the composed variables for individuals with an unsatisfied (“not satisfied”) perception of their neighbourhood did not differ substantially from the mortality hazards obtained for the objective indicators. In other words, no interaction effect was observed for individuals reporting dissatisfaction with the quality of their residential living environment.

However, effect modification was observed for individuals with a satisfied or neutral perception. Among satisfied individuals, we observed that residing in the least optimal objective conditions (highest levels of air pollutants or least surrounding greenness) had the highest mortality hazards. Satisfied individuals living in a poor objective environment may not realize the poor quality of their residential environment. Due to this unawareness, they may see no reason to adopt coping mechanisms (such as visiting nearby parks, paying more attention to other health factors, seeking social support, etc.), which could result in the higher mortality hazards we observed. Ngo et al. (2017) found that mere participation in a study on air pollution led to higher awareness of the relationship between air pollution and health, as well as a more active implementation of coping mechanisms [19]. In this regard, public awareness and citizen science projects (as have recently been launched) should be encouraged as they increase awareness about the quality of one’s living environment.

The beneficial effect of a neutral perception can be interpreted similarly. Individuals with a more neutral perception may be more attuned to the objective conditions of their environment and therefore more likely to adopt coping strategies. This could explain why the relationship between surrounding greenness and all-cause mortality is significantly modified (and even neutralized in the first three quintiles) for individuals with a neutral subjective perception. This aligns with the model of coping and stress by Lazarus (1986) and subsequent adaptations by Lercher (1996) [17,18]. While the objective stressor (e.g., environmental conditions) remains constant, it is the subjective appraisal of this stressor that differs between the groups. Individuals with a neutral perception might be more aware of environmental stressors, thus motivating them to engage in adaptive behaviours.

The question then arises why this effect was not observed among individuals who were not satisfied with the quality of their living environment. One could expect that these individuals would adopt coping mechanisms as well and thus have lower mortality hazards than satisfied individuals. We hypothesize that this might be due to a selection effect. Individuals in poor health might, in general, perceive their neighbourhood more negatively because of both mental and physical challenges. Au et al. (2014) demonstrated that self-assessed health is strongly associated with vitality (energy levels), mobility limits (physical functioning) and bodily pain [26]. These factors may not only influence their perception of their residential environment but also their ability to adopt coping mechanisms, especially when faced with poor conditions.

This interpretation is partly supported by the sensitivity analysis we performed. In the healthy subpopulation—only including individuals assessing their health as “good” or “very good”—individuals with a neutral or even unsatisfied subjective perception exhibited a lower mortality hazard compared to those with a satisfied perception. This suggests that, for healthier individuals, lower satisfaction with the residential environment may indeed lead to the adoption of coping strategies, even among those in the ‘not satisfied’ category. We argue that by focusing on this ‘healthy subpopulation’, we largely exclude individuals who may lack the capacity to adopt effective coping strategies. Furthermore, filtering out individuals in poor health helps mitigate the potential selection effect, where an individual’s health status influences their perception of the residential environment.

## 5. Strengths, Limitations and (Policy) Recommendations

In the discussion above, we hypothesized on the influence of a selection effect, where poor health negatively influences subjective perception. Since we did not dispose of longitudinal data, we could not verify causation between the aforementioned. We partially worked around this limitation by repeating the analysis for a healthy subpopulation. Another option would be to work with aggregated subjective perception instead of individual perception, as demonstrated in other studies [27].

Another limitation is the lack of information on individual lifestyle factors. These factors can influence all-cause mortality [28], but also subjective perception [22] (e.g., someone prone to walking could be more sensitive to the quantity of green spaces in his or her neighbourhood). However, we controlled for socio-economic factors, which have been shown to be related to these individual lifestyle factors [29]. Additionally, we performed a sensitivity analysis for a healthy subpopulation to partially circumvent this limitation. To more effectively address this issue, we We suggest that future studies link with the Health Interview Survey (HIS) conducted by Sciensano, the Belgian national public health body. This survey (although only available for part of the population) would provide detailed information on key lifestyle factors such as smoking, alcohol consumption and physical activity, which could help clarify their role in subjective perception and mortality outcomes. In a similar vein, a more granular distribution of the subjective perception of the residential environment would have been beneficial. Unfortunately, these data were not available.

We could not account for individuals moving from their residential address during follow-up (in our sensitivity analysis, we focussed on individuals who moved before the 2001 census). Also, we did not study the effect of multiple exposures on mortality. To some extent, we may expect some collinearity between the exposure variables (e.g., between green space provision and air quality). But we did not assess what being exposed to, for example, both air and noise pollution meant in comparison with only being exposed to one of these.

A strength of our study is related to our exhaustive dataset combined with a long mortality follow-up period. Additionally, the exposure data were linked to a residential address and had a high spatial resolution. Finally, we could include both subjective and objective indicators of the residential living environment in our analysis. Studies of this kind are relatively scarce and, to our knowledge, had not yet been conducted in Belgium.

Firstly, our study underscores the significant impact of objective indicators (i.e., air and noise pollution and green space availability) on long-term health in the Brussels Capital Region. Given these findings, it is crucial to prioritize actions aimed at improving residential quality across the region. Specifically, the implementation and expansion of green spaces should be a key policy focus, as these not only provide direct health benefits but also help mitigate air and noise pollution. Emerging research suggests that certain types of greenery may be particularly effective in addressing these issues, and further studies should inform policy on this matter.

Secondly, our findings highlight the importance of subjective perception in health outcomes. We have shown that awareness of one’s residential environment plays a central role in how individuals engage with it. Therefore, policy initiatives aimed at raising awareness about environmental quality—through public campaigns, citizen science projects and other forms of engagement—could be highly effective. One recent example of such an initiative is the CurieuzenAir project, which launched in 2021 and involved over 3000 residents measuring air quality at their addresses across Brussels. Expanding such citizen science efforts, alongside more structured public health campaigns, could help foster greater collective awareness, encouraging residents to take proactive steps toward improving their living environment, as well as adopting suitable coping mechanisms.

## Figures and Tables

**Table 1:** Socio-demographic characteristics of the population at baseline (2001), objective and subjective characteristics of the residential living environment and all-cause mortality (2001–2016).

Socio-Demographic Variable	Frequency	Percentage
Gender		
Female	242,953	52.3%
Male	221,658	47.7%
Deaths from all causes during follow-up period 2001–2016		
Died	66,832	14.4%
Emigration during follow-up period 2001–2016		
Emigrated	40,617	8.7%
Highest educational attainment		
Higher education	173,778	37.4%
Secondary education	201,536	43.4%
Primary education or less	89,297	19.2%
Housing tenure		
Owner	224,712	48.4%
Tenant	227,115	48.9%
Other	12,784	2.8%
Household living arrangement		
Couple	285,719	61.5%
Single	172,234	37.1%
Other	6658	1.4%
Migration background		
Other	188,743	40.6%
Belgium	275,868	59.4%
Subjective Perception Variables	Frequency	Percentage
Air quality		
Not pleasant	136,596	29.4%
Satisfactory	266,953	57.5%
Very pleasant	61,062	13.1%
Noise pollution		
Not pleasant	164,525	35.4%
Satisfactory	224,699	48.4%
Very pleasant	75,387	16.2%
Green spaces		
Poorly equipped	113,030	24.3%
Normally equipped	191,770	41.3%
Very well equipped	159,811	34.4%
Objective Variables	Median	Q1–Q3
PM_2.5_ (µg/m^3^) annual average concentration, median (IQR)	19.27	18.95–19.70
NO_2_ (µg/m^3^) annual average concentration, median (IQR)	38.94	35.45–41.72
Daily average noise levels, multiple sources, L_den_ (dB(A))	49.87	47.05–52.92
Surrounding greenness: NDVI 300 m	0.43	0.34–0.53

Source: Belgian 2001 census linked to the mortality register (follow-up 1 October 2001–31 December 2016) and environmental exposure data.

**Table 2:** Hazard ratios (HRs) of all-cause mortality and 95% confidence intervals (95% CIs) for the objective indicators of the residential living environment, BCR, 2001–2016.

Variable	Description	Frequency (%) ^3^	M1 HR (95% CI)	M2 HR (95% CI)
Air pollution: PM_2.5_ (µg/m^3^) annual average concentration ^1^	Q1 (Least exposed)	92,913	1.00 (ref.)	1.00 (ref.)
	Q2	92,830	1.12 ** [1.08; 1.15]	1.05 ** [1.02; 1.09]
	Q3	92,990	1.20 ** [1.16; 1.23]	1.09 ** [1.06; 1.13]
	Q4	92,952	1.23 ** [1.19; 1.27]	1.11 ** [1.08; 1.15]
	Q5 (Most exposed)	92,926	1.28 ** [1.24; 1.32]	1.16 ** 1.12; 1.20]
Air pollution: NO_2_ (µg/m^3^) annual average concentration	Below WHO guideline (40 µg/m^3^)	282,079 (60.7%)	1.00 (ref.)	1.00 (ref.)
	Above WHO guideline	182,532 (39.3%)	1.16 ** [1.13; 1.18]	1.09 ** [1.07; 1.12]
Noise pollution: Multi sources L_den_ (dB(A))	Below WHO guideline (53 dB(A))	350,690 (75.5%)	1.00 (ref.)	1.00 (ref.)
	Above WHO guideline	113,921 (24.5%)	1.04 ** [1.01; 1.06]	1.01 [0.99; 1.04]
Surrounding greenness: NDVI 300 m ^2^	Most surrounding greenness (Q1)	92,920	1.00 (ref.)	1.00 (ref.)
	Q2	92,896	1.09 ** [1.06; 1.12]	1.06 ** [1.03; 1.09]
	Q3	92,952	1.12 ** [1.09; 1.15]	1.06 ** [1.03; 1.09]
	Q4	92,943	1.27 ** [1.23; 1.31]	1.15 ** [1.12; 1.19]
	Least surrounding greenness (Q5)	92,900	1.47 ** [1.42; 1.52]	1.28 ** [1.23; 1.32]

^1^ PM_2.5_ annual average concentration (µg/m^3^) quintiles: quintile 1: [Min; 18.74]; quintile 2: [18.74; 19.10]; quintile 3: [19.10; 19.42]; quintile 4: [19.42; 19.82]; and quintile 5: [19.82; Max]; ^2^ NDVI 300 m surrounding greenness quintiles: quintile 1: [Max; 0.56]; quintile 2: [0.56; 0.46]; quintile 3: [0.46; 0.39]; quintile 4: [0.39; 0.31]; quintile 5: [0.31; Min]. ^3^ Percentages are only displayed when informative. For variables divided into quintiles, all categories are by definition 20.0% and are therefore not shown. ** Significance *p* < 0.01. Results from Cox PH regression models using age as the underlying timescale for the follow-up period 2001–2016. M1 adjusted by gender, M2 = M1 + migrant background, educational level, housing tenure and household living arrangement. Source: Belgian 2001 census linked to the mortality register (follow-up 1 October 2001–31 December 2016) and exposure data.

**Table 3:** Hazard ratios (HRs) of all-cause mortality and 95% confidence intervals (95% CIs) for the subjective perception of the residential living environment, BCR, 2001–2016.

Variable	Description	M1 HR (95% CI)	M2 HR (95% CI)
Perception: Air quality	Very pleasant	1.00 (ref.)	1.00 (ref.)
	Satisfactory	1.00 [0.97; 1.03]	0.97 * [0.94; 0.99]
	Not pleasant	1.11 ** [1.08; 1.15]	1.04 * [1.01; 1.07]
Perception: Noise pollution	Very pleasant	1.00 (ref.)	1.00 (ref.)
	Satisfactory	0.99 [0.97; 1.02]	0.97 * [0.94; 1.00]
	Not pleasant	1.08 ** [1.05; 1.11]	1.01 [0.98; 1.04]
Perception: Green spaces	Very well equipped	1.00 (ref.)	1.00 (ref.)
	Well equipped	1.04 ** [1.02; 1.07]	1.01 [0.98; 1.03]
	Poorly equipped	1.20 ** [1.17; 1.24]	1.10 ** [1.07; 1.14]

* Significance *p* < 0.05. ** Significance *p* < 0.01. Results from Cox PH regression models using age as the underlying timescale for the follow-up period 2001–2016. M1 adjusted by gender, M2 = M1 + migrant background, educational level, housing tenure and household living arrangement. Source: Belgian 2001 census linked to the mortality register (follow-up 1 October 2001–31 December 2016) and exposure data.

## Data Availability

Due to privacy concerns, the data used for this study cannot be made publicly available.

## Supplementary Materials

The following supporting information can be downloaded at https://www.mdpi.com/article/10.3390/ijerph22030391/s1, Tables S1–S3 in the Supplementary Materials show the same results as Figure 1 in the main text. However, the precise numerical values are more easily deducible from the Tables. Table S1 in the Supplementary Materials shows the results (hazard ratios and 95% CIs) for the composed variable on air pollution. Table S2 shows the results for the composed variable on noise pollution, and Table S3 in the Supplementary Materials shows the results for the composed variable on green spaces. Supplementary Tables S4–S6 are part of the sensitivity analysis. They show the results for the same indicators (objective indicators, subjective indicators and composed indicators) under three conditions. The first condition (“Filter 1”) is the same as that used in the main text (i.e., individuals aged 25–79 who have no missing values). The second condition (“Filter 2”) includes only individuals who self-reported their health as either “good” or “very good”. The final condition builds on the first condition but includes only individuals who did not move between 1993 and 2001.
