# Supplementary material for: Perception of the Residential Living Environment: The Relationship Between Objective and Subjective Indicators of the Residential Living Environment and Health
Source: Int J Environ Res Public Health. 2025 Mar 7;22(3):391. doi: 10.3390/ijerph22030391 (PMC11941873; doi:10.3390/ijerph22030391)
Supplement: Supplementary file 1 [file ijerph-22-00391-s001.zip › ijerph-3376721-supplementary.pdf]

Table S1 Hazard ratios (HR) of all-cause mortality and 95% confidence intervals (95% CI) for the composed variables on air pollution, BCR, 2001-2016.

**Variable**	**Description**	**M1** HR (95% CI)	**M2** HR (95% CI)
Subjective perception on ambient air quality/$PM_{2.5}$ concentrations (categorical, µg/m³)	*Satisfied/2nd quintile*	1.26** [1.18;1.35]	1.15** [1.07;1.23]
	Satisfied/3rd quintile	1.33** [1.22;1.44]	1.18** [1.09;1.28]
	Satisfied/4th quintile	1.34** [1.22;1.46]	1.17** [1.07;1.28]
	Satisfied/most polluted quintile	1.44** [1.31;1.58]	1.27** [1.15;1.40]
	Neutral/least polluted quintile	1.04 [0.99;1.10]	1.01 [0.96;1.06]
	Neutral/2nd quintile	1.13** [1.07;1.18]	1.04 [0.99;1.09]
	Neutral/3rd quintile	1.20** [1.14;1.27]	1.07* [1.02;1.123]
	Neutral/4th quintile	1.23** [1.17;1.30]	1.09** [1.04;1.15]
	Neutral/most polluted quintile	1.27** [1.21;1.34]	1.13** [1.07;1.19]
	Not satisfied/least polluted quintile	1.13** [1.05;1.22]	1.04 [0.97;1.12]
	Not satisfied/2nd quintile	1.20** [1.13;1.28]	1.07* [1.01;1.14]
	Not satisfied/3rd quintile	1.31** [1.24;1.39]	1.16** [1.09;1.23]
	Not satisfied/4th quintile	1.34** [1.27;1.42]	1.16** [1.10;1.23]
	Not satisfied/most polluted quintile	1.39** [1.31;1.47]	1.20** [1.14;1.27]
Subjective perception on ambient air quality/NO_2 concentrations (categorical, µg/m³)	*Satisfied/Above WHO threshold*	1.25* [1.17;1.34]	1.14** [1.06;1.21]
	Neutral/Below WHO threshold	1.00 [0.96;1.03]	0.96* [0.93;1.00]
	Neutral/Above WHO threshold	1.13* [1.09;1.18]	1.04* [1.00;1.08]
	Not satisfied/Below WHO threshold	1.09* [1.05;1.14]	1.02 [0.98;1.07]
	Not satisfied/Above WHO threshold	1.23 [1.18;1.28]	1.11** [1.06;1.15]
*Significance p<0.05. **Significance p<0.01. Results from Cox PH regression models using age as the underlying timescale for the follow-up period 2001-2016. M1 adjusted by gender, M2=M1 + migrant background, educational level, housing tenure and household living arrangement.			

Source: Belgian 2001 census linked to the mortality register (follow-up 1st October 2001 – 31st December 2016) and exposure data.

Table S2 Hazard ratios (HR) of all-cause mortality and 95% confidence intervals (95% CI) for the composed variables on noise pollution, BCR, 2001-2016.

**Variable**	**Description**	**M1** HR (95% CI)	**M2** HR (95% CI)
Subjective perception on noise pollution/Noise pollution (multiple sources, L_{den}, categorical) in dB	*Satisfied/Above WHO threshold*	1.12** [1.06;1.19]	1.06 [1.00;1.12]
	Neutral/Below WHO threshold	1.01 [0.97;1.04]	0.97 [0.94;1.01]
	Neutral/Above WHO threshold	1.07** [1.03;1.11]	1.01 [0.97;1.05]
	Not satisfied/Below WHO threshold	1.13** [1.09;1.17]	1.04* [1.00;1.08]
	Not satisfied/Above WHO threshold	1.08** [1.03;1.12]	1.00 [0.96;1.04]
*Significance p<0.05. **Significance p<0.01. Results from Cox PH regression models using age as the underlying timescale for the follow-up period 2001-2016. M1 adjusted by gender, M2=M1 + migrant background, educational level, housing tenure and household living arrangement.			

Source: Belgian 2001 census linked to the mortality register (follow-up 1st October 2001 – 31st December 2016) and exposure data.

Table S3 Hazard ratios (HR) of all-cause mortality and 95% confidence intervals (95% CI) for the composed variables on surrounding greenness, BCR, 2001-2016.

**Variable**	**Description**	**M1** HR (95% CI)	**M2** HR (95% CI)
Subjective perception on green space provision/ NDVI 300m (categorical)	*Satisfied/2nd quintile*	1.10** [1.06;1.15]	1.07** [1.03;1.12]
	Satisfied/3rd quintile	1.11** [1.06;1.16]	1.05* [1.00;1.10]
	Satisfied/4th quintile	1.26** [1.19;1.34]	1.15** [1.08;1.22]
	Satisfied/least surrounding greenness	1.59** [1.47;1.72]	1.34** [1.24;1.45]
	Neutral/most surrounding greeness	0.99 [0.95;1.04]	0.97 [0.93;1.02]
	Neutral/2nd quintile	1.07** [1.03;1.12]	1.02 [0.98;1.07]
	Neutral/3rd quintile	1.10** [1.05;1.14]	1.03 [0.99;1.07]
	Neutral/4th quintile	1.27** [1.22;1.33]	1.14** [1.10;1.19]
	Neutral/least surrounding greenness	1.44** [1.37;1.51]	1.24** [1.18;1.30]
	Not satisfied/most surrounding greeness	1.12** [1.01;1.24]	1.05 [0.95;1.17]
	Not satisfied/2nd quintile	1.17** [1.09;1.26]	1.10** [1.02;1.18]
	Not satisfied/3rd quintile	1.24** [1.16;1.31]	1.14** [1.07;1.21]
	Not satisfied/4th quintile	1.29** [1.22;1.36]	1.15** [1.09;1.21]
	Not satisfied/least surrounding greenness	1.48** [1.41;1.55]	1.27** [1.22;1.34]

*Significance p<0.05. **Significance p<0.01. Results from Cox PH regression models using age as the underlying timescale for the follow-up period 2001-2016. M1 adjusted by gender, M2=M1 + migrant background, educational level, housing tenure and household living arrangement.

Source: Belgian 2001 census linked to the mortality register (follow-up 1st October 2001 – 31st December 2016) and exposure data.

Table S4 Sensitivity analysis - Hazard ratios (HR) of all-cause mortality and 95% confidence intervals (95% CI) for the objective indicators and subjective perception of the residential living environment, BCR, 2001-2016

**Variable**	**Description**	**Filter 1** HR (95%CI)	**Filter 2** HR (95%CI)	**Filter 3** HR (95%CI)
Objective: *Air pollution: $PM_{2.5}$ (µg/m³) annual average concentratio*[1]	Lowest pollution	1.00 (ref.)	1.00 (ref.)	1.00 (ref.)
	2nd quintile	1.05** [1.02;1.09]	1.05 [0.99;1.10]	1.07** [1.03;1.11]
	3rd quintile	1.09** [1.06;1.13]	1.06* [1.01;1.12]	1.10** [1.06;1.14]
	4th quintile	1.11** [1.08;1.15]	1.06 [1.00;1.12]	1.12** [1.08;1.17]
	Highest pollution	1.16** [1.12;1.20]	1.13** [1.07;1.19]	1.16** [1.12;1.21]
Objective: *Air pollution: NO_2 (µg/m³) annual average concentration*	Below WHO guideline (40 *µg/m³*)	1.00 (ref.)	1.00 (ref.)	1.00 (ref.)
	Above WHO guideline	1.09** [1.07;1.12]	1.06** [1.02;1.09]	1.09** [1.07;1.12]
Objective: *Noise pollution: Multi sources L_{den} (dB)*	Below WHO guideline (53 dB)	1.00 (ref.)	1.00 (ref.)	1.00 (ref.)
	Above WHO guideline	1.01 [0.99;1.04]	1.04* [1.00;1.08]	1.00 [0.98;1.04]
Objective: *Surrounding greenness: NDVI 300m*	Most surrounding greenness	1.00 (ref.)	1.00 (ref.)	1.00 (ref.)
	2nd quintile	1.06** [1.03;1.09]	1.08** [1.03;1.13]	1.06** [1.03;1.10]
	3rd quintile	1.06** [1.03;1.09]	1.05* [1.00;1.11]	1.07** [1.03;1.11]
	4th quintile	1.15** [1.12;1.19]	1.10** [1.04;1.16]	1.15** [1.11;1.19]
	Least surrounding greenness	1.28** [1.23;1.32]	1.24** [1.17;1.32]	1.26** [1.21;1.31]
**Subjective:** Perception Air quality	Very Pleasant	1.00 (ref.)	1.00 (ref.)	1.00 (ref.)
	Satisfactory	0.97* [0.94;0.99]	0.88** [0.84;0.93]	0.96* [0.93;1.00]
	Not pleasant	1.04* [1.01;1.07]	0.92** [0.87;0.97]	1.03 [0.99;1.07]
**Subjective:** Perception Noise	Very Pleasant	1.00 (ref.)	1.00 (ref.)	1.00 (ref.)
	Satisfactory	0.97* [0.94;1.00]	0.92** [0.88;0.96]	0.97 [0.94;1.00]
	Not pleasant	1.01 [0.98;1.04]	0.92** [0.88;0.97]	0.99 [0.96;1.03]
**Subjective:** Perception green space	Very well equipped	1.00 (ref.)	1.00 (ref.)	1.00 (ref.)
	Well equipped	1.01 [0.98;1.03]	0.99 [0.95;1.03]	1.01 [0.99;1.04]
	Poorly equipped	1.10** [1.07;1.14]	1.03 [0.98;1.08]	1.10** [1.06;1.14]

*Significance p<0.05. **Significance p<0.01. Results from Cox PH regression models using age as the underlying timescale for the follow-up period 2001-2016. Models adjusted by gender, migrant background, educational level, housing tenure and household living arrangement.

Filter 1: Respondents aged 25-79, who have no missing values on any indicator used. (Results as shown in main article)
Filter 2: Filter 1 + Self-assessed health is "good" or "very good".
Filter 3: Filter 1 + the respondents did not move between 1991 and 2003

Source: Belgian 2001 census linked to the mortality register (follow-up 1st October 2001 – 31st December 2016) and exposure data.

Table S5 Sensitivity analysis - Hazard ratios (HR) of all-cause mortality and 95% confidence intervals (95% CI) for the composed variables of the residential living environment (part 1), BCR, 2001-2016

**Variable**	**Description**	**Filter 1** HR (95%CI)	**Filter 2** HR (95% CI)	**Filter 3** HR (95%CI)
Composed: *Subjective perception on ambient air quality/$PM_{2.5}$ concentrations (categorical, µg/m³)*	*Satisfied/least polluted*	1.00 (ref.)	1.00 (ref.)	1.00 (ref.)
	Satisfied/2nd quintile	1.15** [1.07;1.23]	1.19** [1.08;1.32]	1.21** [1.12;1.31]
	Satisfied/3rd quintile	1.18** [1.09;1.28]	1.08 [0.95;1.22]	1.22** [1.11;1.34]
	Satisfied/4th quintile	1.17** [1.07;1.28]	1.20** [1.05;1.38]	1.15** [1.03;1.28]
	Satisfied/most polluted quintile	1.27** [1.15;1.40]	1.33** [1.15;1.53]	1.31** [1.16;1.46]
	Neutral/least polluted quintile	1.01 [0.96;1.06]	0.93* [0.86;1.00]	1.01 [0.96;1.07]
	Neutral/2nd quintile	1.04 [0.99;1.09]	0.95 [0.88;1.02]	1.05 [1.00;1.11]
	Neutral/3rd quintile	1.07* [1.02;1.12]	0.98 [0.91;1.06]	1.08* [1.02;1.14]
	Neutral/4th quintile	1.09** [1.04;1.15]	1.00 [0.93;1.09]	1.12** [1.05;1.19]
	Neutral/most polluted quintile	1.13** [1.07;1.19]	1.03 [0.95;1.12]	1.14** [1.07;1.21]
	Not satisfied/least polluted quintile	1.04 [0.97;1.12]	0.94 [0.83;1.06]	1.05 [0.96;1.14]
	Not satisfied/2nd quintile	1.07* [1.01;1.14]	0.98 [0.89;1.09]	1.09* [1.02;1.17]
	Not satisfied/3rd quintile	1.16** [1.09;1.23]	1.05 [0.95;1.16]	1.16** [1.09;1.25]
	Not satisfied/4th quintile	1.16** [1.10;1.23]	0.94 [0.85;1.04]	1.17** [1.10;1.25]
	Not satisfied/most polluted quintile	1.20** [1.14;1.27]	1.07 [0.98;1.18]	1.21** [1.13;1.22]
Composed: *Subjective perception on ambient air quality/NO_2 concentrations (categorical, µg/m³)*	*Satisfied/Below WHO threshold*	1.00 (ref.)	1.00 (ref.)	1.00 (ref.)
	Satisfied/Above WHO threshold	1.14** [1.06;1.21]	1.18** [1.06;1.30]	1.12** [1.04;1.22]
	Neutral/Below WHO threshold	0.96* [0.93;1.00]	0.89** [0.85;0.94]	0.96* [0.92:1.00]
	Neutral/Above WHO threshold	1.04* [1.00;1.08]	0.96 [0.90;1.01]	1.04 [1.00;1.09]
	Not satisfied/Below WHO threshold	1.02 [0.98;1.07]	0.94 [0.88;1.00]	1.01 [0.97;1.06]
	Not satisfied/Above WHO threshold	1.11** [1.06;1.15]	0.95 [0.89;1.01]	1.10 [1.05;1.15]

*Significance $p<0.05$. **Significance $p<0.01$. Results from Cox PH regression models using age as the underlying timescale for the follow-up period 2001-2016. Models adjusted by gender, migrant background, educational level, housing tenure and household living arrangement.

Filter 1: Respondents aged 25-79, who have no missing values on any indicator used. (Results as shown in main article)
Filter 2: Filter 1 + Self-assessed health is “good” or “very good”.
Filter 3: Filter 1 + the respondents did not move between 1991 and 2003

Source*:* Belgian 2001 census linked to the mortality register (follow-up 1st October 2001 – 31st December 2016) and exposure data.

Table S6 Sensitivity analysis - Hazard ratios (HR) of all-cause mortality and 95% confidence intervals (95% CI) for the composed variables of the residential living environment (part 2), BCR, 2001-2016

**Variable**	**Description**	**Filter 1** HR (95%CI)	**Filter 2** HR (95% CI)	**Filter 3** HR (95%CI)
Composed: *Subjective perception on green space provision/ NDVI 300m (categorical)*	*Satisfied/most green space*	1.00 (ref.)	1.00 (ref.)	1.00 (ref.)
	Satisfied/2nd quintile	1.07** [1.03;1.12]	1.08* [1.02;1.16]	1.08** [1.03;1.13]
	Satisfied/3rd quintile	1.05* [1.00;1.10]	1.08 [1.00;1.16]	1.05 [0.99;1.11]
	Satisfied/4th quintile	1.15** [1.08;1.22]	1.05 [0.95;1.16]	1.15** [1.07;1.23]
	Satisfied/least green space	1.34** [1.24;1.45]	1.34** [1.17;1.53]	1.26** [1.14;1.39]
	Neutral/most green space	0.97 [0.93;1.02]	0.97 [0.90;1.04]	0.98 [0.93;1.03]
	Neutral/2nd quintile	1.02 [0.98;1.07]	1.01 [0.95;1.08]	1.03 [0.98;1.08]
	Neutral/3rd quintile	1.03 [0.99;1.07]	1.00 [0.93;1.07]	1.04 [1.00;1.09]
	Neutral/4th quintile	1.14** [1.10;1.19]	1.12** [1.04;1.20]	1.15** [1.09;1.21]
	Neutral/least green space	1.24** [1.18;1.30]	1.25** [1.15;1.36]	1.26** [1.19;1.34]
	Not satisfied/most green space	1.05 [0.95;1.17]	0.90 [0.74;1.09]	1.08 [0.96;1.21]
	Not satisfied/2nd quintile	1.10** [1.02;1.18]	1.17* [1.03;1.32]	1.11* [1.02;1.21]
	Not satisfied/3rd quintile	1.14** [1.07;1.21]	1.08* [0.97;1.20]	1.16** [1.08;1.25]
	Not satisfied/4th quintile	1.15** [1.09;1.21]	1.04 [0.95;1.14]	1.14** [1.07;1.21]
	Not satisfied/least green space	1.27** [1.22;1.34]	1.16** [1.07;1.26]	1.25** [1.18;1.32]
Composed: *Subjective perception on noise pollution/Noise pollution (multiple sources, L_{den}, categorical) in dB*	*Satisfied/Below WHO threshold*	1.00 (ref.)	1.00 (ref.)	1.00 (ref.)
	Satisfied/Above WHO threshold	1.06 [1.00;1.12]	1.09 [1.00;1.19]	1.07 [1.00;1.14]
	Neutral/Below WHO threshold	0.97 [0.94;1.01]	0.92** [0.88;0.97]	0.98 [0.94;1.02]
	Neutral/Above WHO threshold	1.01 [0.97;1.05]	0.99 [0.93;1.06]	1.00 [0.95;1.05]
	Not satisfied/Below WHO threshold	1.04* [1.00;1.08]	0.95 [0.90;1.01]	1.02 [0.98;1.06]
	Not satisfied/Above WHO threshold	1.00 [0.96;1.04]	0.93 [0.86;1.00]	0.99 [0.94;1.04]

*Significance p<0.05. **Significance p<0.01. Results from Cox PH regression models using age as the underlying timescale for the follow-up period 2001-2016. Models adjusted by gender, migrant background, educational level, housing tenure and household living arrangement.

Filter 1: Respondents aged 25-79, who have no missing values on any indicator used. (Results as shown in main article)
Filter 2: Filter 1 + Self-assessed health is "good" or "very good".
Filter 3: Filter 1 + the respondents did not move between 1991 and 2003

Source: Belgian 2001 census linked to the mortality register (follow-up 1st October 2001 – 31st December 2016) and exposure data.
